# Visualizing Research Trends in Quantum Dots for Health: A Bibliometric Exploration

**DOI:** 10.7759/cureus.70132

**Published:** 2024-09-24

**Authors:** Remya Krishnan M, Sreya P, Shaija P.B, Saiju D.I, Jobin Jose

**Affiliations:** 1 Department of Physics, Sree Narayana College, Kannur, IND; 2 Department of Chemistry, Sree Narayana College, Kannur, IND; 3 Department of Chemistry, Sree Narayana College, Cherthala, IND; 4 Department of Library Science, Sree Narayana College, Kollam, IND; 5 Department of Library Science, Marian College Kuttikkanam (Autonomous), Kuttikkanam, IND

**Keywords:** bibliometric analysis, biblioshiny, health, quantum dots, vosviewer

## Abstract

Quantum dots (QDs) are semiconductor nanoparticles with immense potential accompanied by unique optoelectronic properties for revolutionizing several biomedical applications related to imaging, diagnostics, drug delivery, and therapy. A detailed bibliometric analysis has been performed in this article with regard to research relating to QDs in the health sector during 2004-2024. In spite of their promising applications, assessment of the toxicity of QDs, especially highly toxic heavy metal-based QDs like cadmium, is still the most important apprehension. This analysis identifies from Scopus data key trends, influential authors, leading sources, and significant collaborative networks in the field. On VOSviewer and Biblioshiny, visualization tools are used to show research trends and networks that help in the discovery of critical knowledge about the entire global landscape of research. The increasing scientific interest has been realized in a stable trend with peaks of notable and emerging topics attesting to its dynamism. The study epitomizes how international collaboration can advance the QD applications boundary for healthcare.

## Introduction and background

Quantum dots (QDs) are semiconductor nanoparticles with unique optoelectronic properties due to quantum confinement effects. Small in size and likely to have their optical properties tuned, they come into a wide scope of biomedical applications, from imaging and diagnosis to drug delivery and therapy. QDs find broad application in biomedical imaging because they are highly bright and resistant to photobleaching, and their emission spectra can be changed by varying the size and composition. They work well in applications involving real-time tissue imaging, bioimaging, and single-molecule probes. Such capabilities make them very useful in diagnostics like early cancer detection and in vivo imaging of tumors [1,2].

QDs have already shown high potential for drug delivery due to the possibility of being functionalized with regard to targeted delivery. They transport drugs directly into cells or tissues, hence reducing side effects and increasing therapeutic outcomes. Theranostics in cancer treatment are especially very promising fields of application because they help simultaneously image and treat tumors [3,4]. QDs have also been explored for their potential in treating various diseases. They have been proposed as a new modality for the treatment of cancer and neurodegenerative disorders such as Alzheimer's and Parkinson's. The fact that they are able to cross the blood-brain barrier to deal with central nervous system disorders is a remarkable development that opened new avenues for the treatment of such conditions [5,6].

Though there exist very promising applications, the toxicity of QDs is still a critical concern, especially due to the presence of heavy metals like cadmium in their core. Several attempts have been made to decrease the toxicity by surface modification. Biocompatible QDs were fabricated, among them graphene QDs that showed lower cytotoxicity and higher biocompatibility [7,8]. The development of QDs today faces continued challenges in improving their biocompatibility and ensuring their efficient body clearance. Improvements in molecular design for QDs involve better long-term stability in biological buffers and higher quantum yield after bioconjugation. All these efforts are very important in ensuring the safe and effective clinical translation of QDs [1]. QDs are one of the most versatile and powerful tools in modern medicine today, from diagnostics to targeted therapy; in fact, the QD ushered in a new frontier in medical research and application. Though challenges with their toxicity and biocompatibility remain, ongoing research and development efforts pave the way toward their broader clinical application. Continued innovation in this field will unlock even greater potential for QDs in improving human health [9].

Bibliometric analysis is a quantitative approach toward the evaluation of research impact and trends by publication patterns, citation data, and collaboration network analysis [10-14]. Two main tools in this area are VOSviewer and Biblioshiny for RStudio [15,16]. VOSviewer has the advantage of constructing and visualizing bibliometric networks like co-authorship and citation networks. It provides researchers with a means to visualize a connection or trend within vast data [17-20]. On the other hand, Biblioshiny is a web application designed to analyze trends of publications and citation patterns in the Bibliometrix package itself within RStudio, hence user-friendly for those not having any programming background [21-24]. These tools, when put together, will go a long way in offering comprehensiveness and visualization in the analysis of scientific literature, hence unveiling the important publications and topics in the field of research.

The aim of this study is to provide a comprehensive overview of the research landscape surrounding QDs in the health sector. By utilizing bibliometric analysis, the study seeks to identify key publication trends, major research themes, leading contributors, and prominent countries in the field. Additionally, it aims to map out collaborative networks and thematic developments over the past two decades, highlighting emerging topics and ongoing challenges, such as toxicity concerns. Through this, the study intends to offer insights into the current state of QD research in biomedical applications and identify potential future research directions.

## Review

Materials and methods

**Figure 1:**
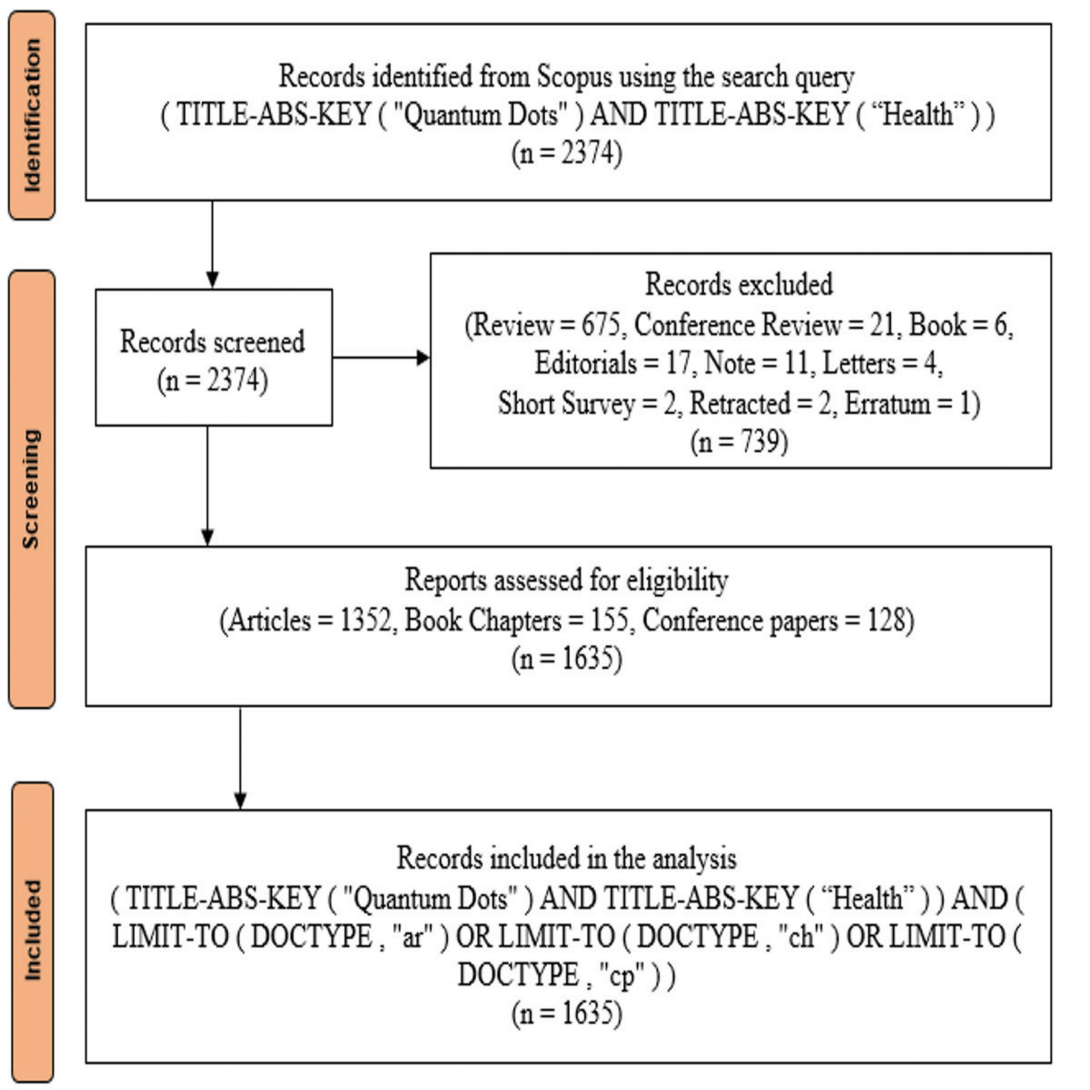
PRISMA Flowchart PRISMA: Preferred Reporting Items for Systematic Reviews and Meta-Analyses

In this study, Scopus was deliberately chosen as the primary source of bibliographic data due to its extensive and diverse collection of high-quality academic journals, offering a broader and more comprehensive coverage compared to other available databases. Scopus is a reliable and comprehensive database, and therefore it was selected to capture the maximum range of research products in many disciplines. Publications were sought using the keywords "Quantum Dots" AND "health", with no restriction on languages. This allowed for the investigation of studies relevant to the topic from different linguistic-cultural backgrounds, increasing the global relevance of the analysis. In particular, journal articles, book chapters, and conference papers were targeted in this research because such publications generally reflect serious contributions to the field. In this period of twenty years-from 2004 to 2024-the search came up with 1,635 documents from 641 sources. This large dataset served as a sound basis for carrying out a comprehensive bibliometric analysis. Figure 1 illustrates the Preferred Reporting Items for Systematic Reviews and Meta-Analyses (PRISMA) methodology, a systematic approach applied for the phase of identification, screening, and selection of papers included in the bibliometric analysis. This study broke down the PRISMA process into three steps: The first step of identification extracted data related to relevant research from the Scopus database, where all types of documents matching the initial search criteria were collected. The first step was done to make the dataset as inclusive as possible before further refining it. The second step separated those publications that were less core to the research objectives. In particular, reviews, editorials, letters, notes, and short surveys were excluded because usually no original research results are reported in these formats. The selection was thus narrowed to include only Articles, Book Chapters, and Conference Papers-forms generally recognized as making substantive contributions to scholarly discourse. Ensuring that only quality publications remained in the dataset was important for appropriate conduct of the analysis to be meaningful and focused on leading-edge research artifacts. This refined dataset was then saved as a comma-separated values (CSV) file for use in the bibliometric analysis. The tools used for the analysis were two robust software packages: VOSviewer and Biblioshiny. While interactive, both tools allowed for a full and intuitively clear analysis of the scientific literature with respect to identifying influential works, mapping themes of research development, and emerging areas of interest related to QDs in health. This research methodological rigor was only possible with the help of advanced bibliometric tools, providing deep and nuanced insight into the research landscape and driving enormous value within the field.

Table 1 summarizes the main results of the bibliometric analysis of research dealing with QDs and health within the time period from 2004 to 2024. The documents retrieved correspond to 1,635 publications in 641 journals, books, or other sources. According to this thematic area, it has a low annual growth rate of 3.36%. The average document age is approximately 4.7 years. At an average of 24.03 citations per document, it showcases the seriousness of the impact and applicability of this research area, which, in total, covers 85,868 references by all documents. Looking at this content-wise, there were a total of 13,343 keywords plus and 4,150 author's keywords, pointing to a wide variety of issues argued within this field. Furthermore, the researchers have highlighted the intense level of collaboration among themselves, with 5,884 authors contributing to the body of work. Importantly, out of these, only 54 authors were found to produce single-authored documents, and most of the research was collaborative in nature, as shown by an average of 5.99 co-authors per document. Besides this, international cooperation was found in 19.63% of the studies; this proves that this area of research has international dimensions. In terms of document types, the bulk of the output consists of journal articles at 1,352, followed by book chapters at 155 and then conference papers at 128. This distribution reflects emphasis on the dissemination of research findings through peer-reviewed articles while simultaneously contributing toward scholarly books and conferences. Most of the features are put together to provide an overview of the research activity and collaborative efforts in the study of QDs in health.

**Table 1:** Main Information of the Investigation

Description	Results
Timespan (years)	2004:2024
Sources (journals, books, etc.)	641 sources
Documents	1635 documents
Annual growth rate %	3.36% per year
Document average age (years)	4.7 years
Average citations per doc	24.03 citations per document
References	85868 references
Document contents	
Keywords plus (ID)	13343 keywords
Author's keywords (DE)	4150 keywords
Authors	
Authors	5884 authors
Authors of single-authored docs	54 authors
Authors collaboration	
Single-authored docs	56 documents
Co-Authors per doc	5.99 authors per document
International co-authorships %	19.63%
Document types	
Article	1352 articles
Book chapter	155 book chapters
Conference paper	128 conference papers

Annual Scientific Production

**Figure 2:**
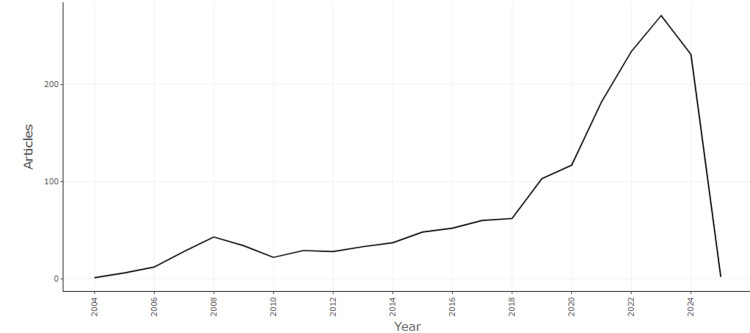
Annual Scientific Production

Figure 2 illustrates the annual publication trend of articles related to QDs in health from 2004 to 2024. The graph shows a gradual increase in the number of articles from 2004, with a noticeable peak occurring around 2022. This peak represents the highest volume of publications within the 20-year span, indicating a significant surge in research interest and activity during this period. After 2022, there is a sharp decline in the number of articles, with 2024 seeing a dramatic drop. This decline may suggest several possibilities, including a shift in research focus, saturation in the field, or external factors such as changes in funding or publication practices. The trend highlights the dynamic nature of research in this area, with a clear rise in interest and subsequent decline, which may warrant further investigation into the causes and implications of these shifts.

Most Relevant Authors

Table 2 highlights the most prolific authors in the field of QDs in health, as indicated by the number of articles they have published. The data reveals that Wang Y is the most active author, contributing to 58 articles, followed closely by Liu Y with 52 articles and Li Y with 50 articles. Zhang Y and Zhang J are also prominent contributors, with 49 and 44 articles, respectively. Additionally, authors such as Liu J, Wang L, and Wang X have each published 40 articles, demonstrating a significant presence in the field. Wang J and Wang S round out the list with 39 and 33 articles, respectively. The prevalence of these authors indicates their leading roles in advancing research on QDs in health, reflecting their influence and consistent contributions to the academic discourse in this area.

**Table 2:** Most Relevant Authors

Authors	Articles
Wang Y	58
Liu Y	52
Li Y	50
Zhang Y	49
Zhang J	44
Liu J	40
Wang L	40
Wang X	40
Wang J	39
Wang S	33

Most Relevant Sources

Table 3 presents the most relevant sources in the field of QDs in health, based on the number of articles published. Food Chemistry emerges as the leading journal, with 47 articles, indicating its significant role in disseminating research in this area. Analytica Chimica Acta follows with 37 articles, and Spectrochimica Acta - Part A: Molecular and Biomolecular Spectroscopy has contributed 32 articles, showcasing their importance in the field. Other notable sources include the Journal of Hazardous Materials with 26 articles and Sensors and Actuators B: Chemical with 25 articles. RSC Advances (24 articles), Journal of Nanoparticle Research (22 articles), Microchemical Journal (22 articles), Talanta (22 articles), and Biosensors and Bioelectronics (21 articles) also play key roles in publishing research on QDs in health. These journals are critical platforms for the ongoing exploration and dissemination of knowledge in this rapidly evolving area of study.

**Table 3:** Most Relevant Sources

Sources	Articles
Food Chemistry	47
Analytica Chimica Acta	37
Spectrochimica Acta - Part A: Molecular and Biomolecular Spectroscopy	32
Journal of Hazardous Materials	26
Sensors and Actuators B: Chemical	25
RSC Advances	24
Journal of Nanoparticle Research	22
Microchemical Journal	22
Talanta	22
Biosensors and Bioelectronics	21

Countries' Scientific Production

Table 4 provides an overview of the countries contributing the most to scientific production in the field of QDs in health. China leads by a significant margin, with 4,912 articles, reflecting its dominant position and substantial investment in this area of research. The USA follows with 1,069 articles, demonstrating strong research activity and influence. India is also a key player, contributing 947 articles, highlighting its growing focus on this field. Other countries with notable contributions include South Korea with 268 articles, and Brazil with 227 articles, indicating active research communities in these regions. Iran (221 articles) and Italy (163 articles) also make significant contributions. Canada (131 articles), the UK (120 articles), and Australia (105 articles) round out the top contributors, each playing a crucial role in advancing research on QDs in health. The data underscores the global nature of research in this field, with substantial contributions coming from both developed and developing countries.

**Table 4:** Countries' Scientific Production

Country	Number of articles
China	4912
USA	1069
India	947
South Korea	268
Brazil	227
Iran	221
Italy	163
Canada	131
UK	120
Australia	105

Trend Topics

**Figure 3:**
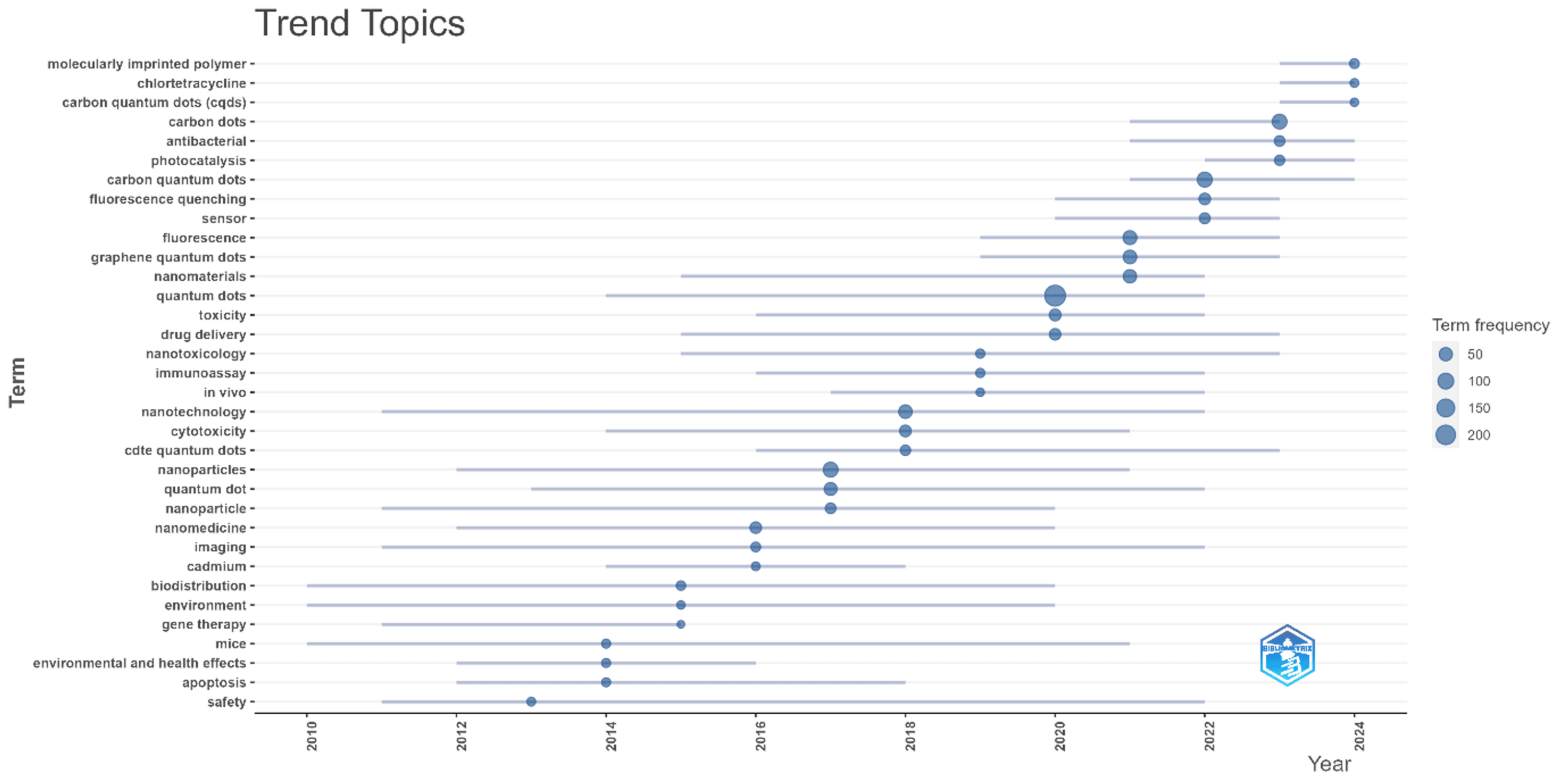
Trend Topics

Figure 3 presents the trend topics in research related to QDs in health over time, illustrating how the frequency of specific terms has evolved from 2012 to 2024. The horizontal axis represents the years, while the vertical axis lists various research terms associated with QDs. The size of the circles corresponds to the frequency of each term, with larger circles indicating more frequent usage. The figure shows that topics such as "molecularly imprinted polymer," "chlorine/triclosan," and "carbon quantum dots" have gained prominence in recent years, particularly after 2020. This suggests a growing research interest in these areas. Other terms like "fluorescence," "graphene quantum dots," and "quantum dots" have seen consistent usage across the years, indicating their continued relevance in the field. Emerging topics like "biosensors," "nanotechnology," and "drug delivery" have also become more frequent in publications, reflecting the expanding applications of QDs in health-related research. The trend toward interdisciplinary applications is evident in terms like "environmental and health effects," "apoptosis," and "safety," which have become more prominent, particularly in the latter part of the period covered. Overall, this highlights the dynamic nature of research on QDs in health, with evolving trends that reflect both the maturation of the field and the exploration of new applications and technologies. The visualization effectively captures the shifts in research focus over time, providing insights into the direction of future studies.

Thematic Map

**Figure 4:**
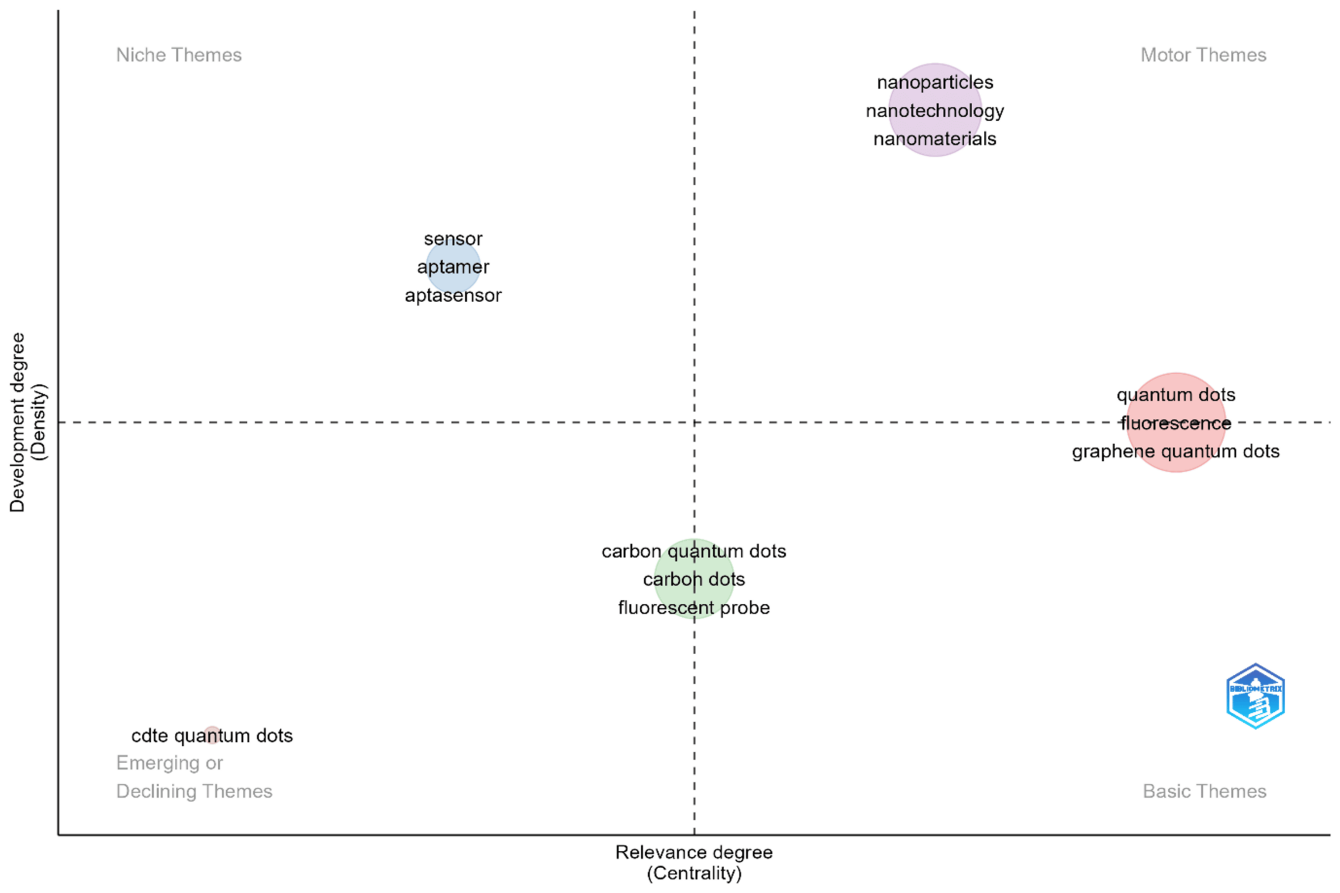
Thematic Map

Figure 4 illustrates a thematic map that categorizes research themes related to QDs in health, providing insight into their development (density) and relevance (centrality). The map is divided into four quadrants, each representing different types of themes within the research landscape. In the Motor Themes quadrant (upper right), we find highly developed and central themes that are driving research in the field. Topics such as "nanoparticles," "nanotechnology," and "nanomaterials" are located here, indicating that these areas are not only well-established but also pivotal to advancements in QDs and health. These themes are likely leading innovation and are the focus of significant scholarly attention, making them critical to the ongoing development of the field. The Basic Themes quadrant (lower right) includes fundamental but perhaps less developed themes that are nonetheless central to the research domain. In this area, "quantum dots," "fluorescence," and "graphene quantum dots" are key topics. These themes form the foundation upon which much of the research is built, providing essential concepts and methodologies that support more specialized studies. Despite being foundational, these themes continue to be of great relevance, contributing to the broader understanding and application of QDs in health. The Niche Themes quadrant (upper left) contains specialized topics that are highly developed but less central to the broader field. Terms like "sensor," "aptamer," and "aptasensor" fall into this category. These themes represent areas of intense research within narrower scopes, often involving cutting-edge technologies or highly specific applications. While these topics may not be as central as those in the motor themes, their high development indicates that they are of significant interest within their specific niches. Finally, the Emerging or Declining Themes quadrant (lower left) features themes that are less developed and less central. The term "CdTe quantum dots" is an example in this quadrant, suggesting that research in this area might be in decline or is only beginning to emerge. These themes may represent new frontiers that have yet to gain widespread attention or areas where research interest is waning. Overall, it provides a comprehensive overview of the thematic structure in the field of QDs in health. By mapping the development and relevance of various research themes, it offers valuable insights into the current state of the field, highlighting both well-established areas and potential avenues for future exploration.

Collaboration Network

**Figure 5:**
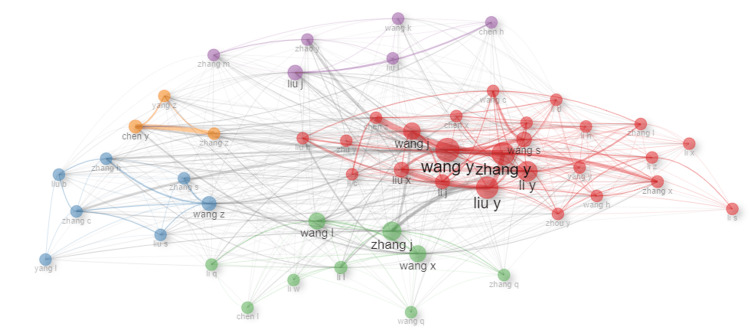
Collaboration Network Among Authors

Figure 5 depicts a collaboration network among authors who have contributed to research on QDs in health. The nodes in the network represent individual authors, while the edges (lines) between them indicate collaborative relationships based on co-authorship. The size of each node reflects the number of publications by the respective author, and the thickness of the edges represents the strength of collaboration, with thicker lines indicating more frequent co-authorship.

The network reveals several clusters of tightly knit collaborations, suggesting the presence of research teams or groups that frequently work together. Notably, the central cluster is dominated by authors like Wang Y, Zhang Y, and Liu Y, who appear to be the most prolific and central figures in this research domain, as indicated by the larger node sizes. These authors are connected by numerous collaborations, highlighting their influential roles in advancing the field.

Other clusters in the network, represented by different colors, indicate additional collaborative groups. For instance, authors such as Chen Y, Yang Z, and Zhang S form a distinct cluster, suggesting that they collaborate closely within a specific subfield of QD research. Similarly, the presence of smaller and more isolated clusters suggests specialized groups that may focus on niche areas within the broader research field.

Overall, the collaboration network illustrates the collaborative nature of research on QDs in health, emphasizing the key contributors and the interconnectedness of different research groups. The network visualization helps identify the most influential researchers and the collaborative structures that drive the field forward.

Co-authorship Between Countries

**Figure 6:**
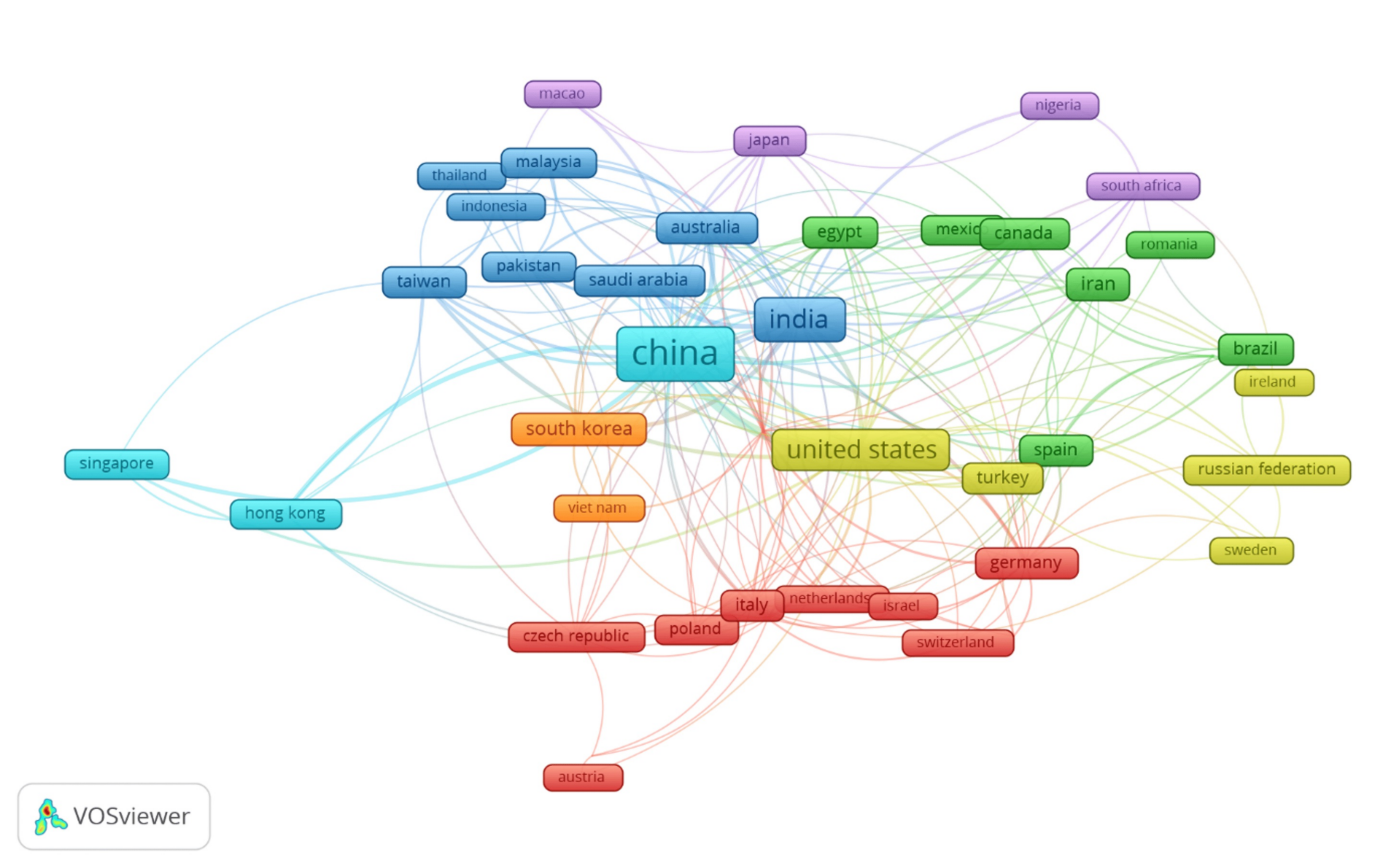
Co-authorship Between Countries

Figure 6 illustrates the co-authorship network between countries involved in research on QDs in health. Each node represents a country, and the size of the node corresponds to the number of publications attributed to that country. The lines connecting the nodes indicate collaborative relationships, with thicker lines representing stronger or more frequent collaborations between countries. The network shows that China, India, and the United States are the most prominent and central countries in this research area, as indicated by the larger node sizes. These countries have numerous connections with other nations, reflecting their strong international collaboration networks. China appears to be particularly central, with extensive links to countries across Asia, Europe, and North America, indicating its pivotal role in global research collaborations on QDs in health. India and the United States also exhibit substantial collaboration networks, with significant connections to countries like Germany, Italy, and South Korea. These countries form key hubs in the global research landscape, contributing to a large portion of the scientific output in this field.

Other countries such as Germany, Italy, and South Korea are also well-connected, suggesting active participation in international collaborations. The presence of connections between European countries (e.g., Germany, Italy, Netherlands) and between countries in Asia (e.g., China, Japan, South Korea) highlights the regional collaboration patterns that exist within the broader global network. The map also reveals clusters of countries that frequently collaborate with one another, such as the collaboration between Australia and Malaysia, and between Brazil and Spain, which suggests regional or thematic partnerships. Overall, the co-authorship pattern underscores the global and collaborative nature of research on QDs in health, with major contributions coming from a few key countries that are well-integrated into international research networks. This visualization highlights the importance of cross-border collaborations in advancing scientific knowledge and innovation in this rapidly evolving field.

Co-occurrence of Keywords

**Figure 7:**
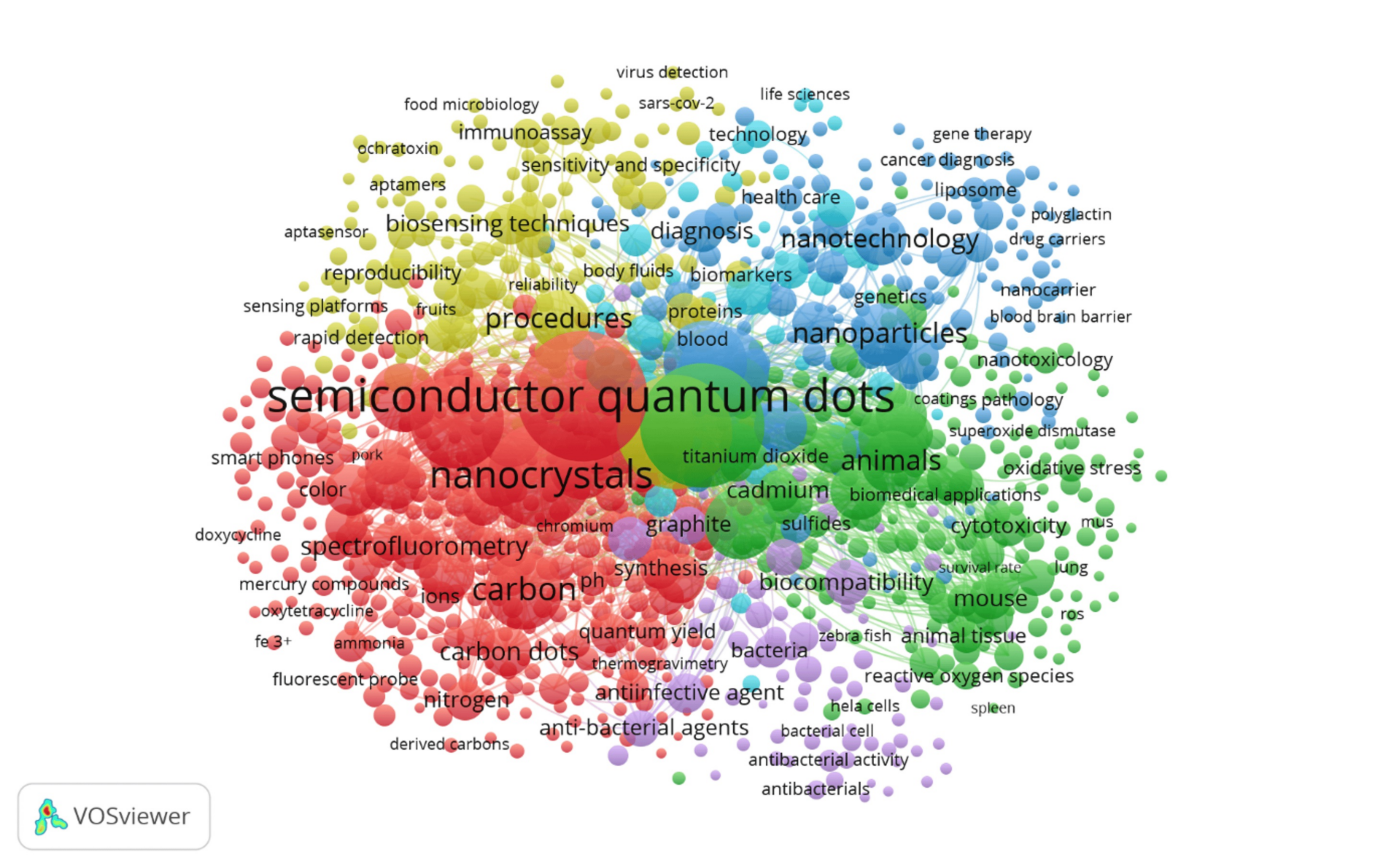
Co-occurrence of Keywords

Figure 7 illustrates the co-occurrence of keywords in research related to QDs in health, revealing the interconnectedness and thematic organization of the field. The map features several distinct clusters, each representing a major area of research. The red cluster is centered around "semiconductor quantum dots" and "nanocrystals," indicating that these are core topics in the field, with a strong emphasis on synthesis, characterization, and applications, particularly in areas like "spectrofluorometry" and "carbon quantum dots." The green cluster focuses on "biocompatibility," "nanoparticles," and "animals," highlighting research on the biological and medical implications of QDs, including their safety and interactions within biological systems. The blue cluster emphasizes "nanotechnology," "nanoparticles," and "biomarkers," reflecting the integration of QDs into broader nanotechnological applications, particularly in diagnostics and targeted therapy, with keywords like "gene therapy" and "drug carriers" also playing a significant role. The yellow cluster centers on "biosensing techniques," "diagnosis," and "immunoassay," showcasing the use of QDs in developing advanced diagnostic tools and biosensors, with applications extending to fields such as "food microbiology" and "virus detection." This map, with its densely populated nodes and connections, provides a comprehensive view of the interconnected research themes in QDs, highlighting both well-established areas and emerging trends, thus offering valuable insights into the current state and potential future directions of the field.

Bibliographic Coupling Between Countries

**Figure 8:**
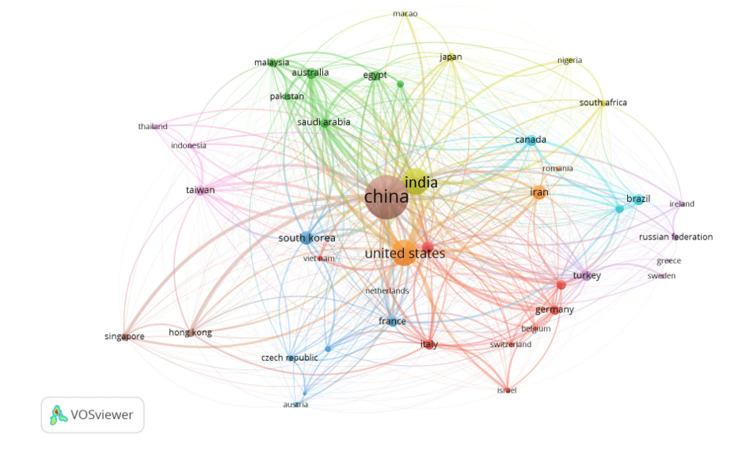
Bibliographic Coupling Between Countries

Figure 8 visualizes the bibliographic coupling between countries in research related to QDs in health. Bibliographic coupling occurs when two countries cite the same third country in their research, indicating a shared foundation or influence in their scientific work. In this network, each node represents a country, and the size of the node corresponds to the volume of research output. The lines (edges) connecting the nodes indicate the strength of the bibliographic coupling, with thicker lines representing stronger connections. The map shows that China and the United States are the central hubs in this network, with the largest nodes and the most connections, indicating their dominant roles in the global research landscape on QDs. China appears to be particularly influential, with strong bibliographic coupling to numerous countries across different continents, suggesting that its research is frequently cited and serves as a key reference point for other nations.

Other countries with significant bibliographic coupling include Germany, South Korea, and France, each of which has numerous connections, reflecting their active participation and influence in the global research community. European countries like Germany, Italy, and France are notably interconnected, indicating strong regional collaboration and shared research foundations. The network also highlights the involvement of countries from different regions, such as Canada, Iran, Saudi Arabia, and Brazil, which, despite being smaller nodes, play crucial roles in linking various parts of the global research community. These countries contribute to the diversity of perspectives and research approaches in the field. Overall, this underscores the collaborative nature of QDs research, with countries like China and the United States at the core, influencing and being influenced by a wide array of international research efforts. The network visualization provides insights into the global distribution of research influence, showing how different countries are interconnected through their shared bibliographic references, ultimately contributing to the collective advancement of knowledge in this field.

Discussion

This bibliometric analysis of health research into QDs accounts for the important evolvement occurring in this field during the last two decades. In the wake of this, one can see a gradual increase in publications reaching a peak around 2022, setting a tone for growing interest in biomedical applications of QDs. If this peak occurred in 2022, followed by a sensible decrease in 2024, it might have indicated shifting interests due to new technologies, scientific priorities, and toxic issues related to QDs [4]. The noticeable decrease in publications in 2024 could be attributed to the emergence of new technologies and a shift in scientific priorities, diverting attention from QDs. Additionally, unresolved toxicity issues related to QDs may have led to reduced research output as concerns over their safety and environmental impact gained prominence. To this end, the identification of key contributors, China, and the United States, and India, highlights international collaboration in this field. Moreover, the most productive journals and authors reflect how particular research groups and regions have concentrated efforts and become leading actors in the research of QDs in health.

Unique optoelectronic properties in the line of bright luminescence, photostability, and tunable emission spectra of QDs spur biomedical imaging and diagnostics. Such features enable huge advances in real-time tissue imaging, bioimaging, and even probing single molecules, which secure a place for QDs as particularly useful for in vivo imaging of tumors and noninvasive early cancer detection [1]. However, QD toxicity remains a harrowing challenge, especially that resulting from the presence of heavy metals such as cadmium. This is the critical barrier to their broad application in a clinical setting. While there have been advances concerning the reduction of toxicity through surface modification and the development of biocompatible QD alternatives such as graphene QDs, the safe and effective application of QDs in medical treatment remains a large area of concern and research [25].

Literature thematic analysis thus reflects emergent research trends, such as growing interest in biocompatibility, biosensors, and targeted drug delivery. It can be seen as one direction of maturation of the field: from general exploratory studies toward more specialized application-driven research. Fluorescence and nanoparticles remain the core topics in the field, but some new areas, mainly related to theranostics and personalized medicine, are becoming prominent [26]. This analysis basically results in a definition of how the scientific community is increasingly exploring QDs more beyond traditional imaging and diagnostics, moving toward larger, more integrative, and therapeutic applications.

Research Gaps and Future Directions

Despite the huge amount of literature on QDs in health, some critical gaps still remain, thus opening up a host of opportunities for future exploitation. The most important ones pertain to the toxicity and biocompatibility of QDs. While progress has been made in lowering the toxicity through surface engineering and the development of other alternatives like GQDs, further studies are needed to comprehend the long-term effects QDs have on living organisms [27]. Future studies should focus on the development of less toxic, more biocompatible, and efficiently system-cleared QDs to ensure their safe use in medical applications.

Another key future research direction is the clinical translation of QDs. Despite the enormous potential QDs have had in a laboratory study, they are met with a slow transition to real-world applications in clinics [28]. Efforts are required to bridge the gap by increasing clinical trials, optimization of QDs for intended medical applications, and addressing the regulatory challenges that need to be cleared for use in humans. It will have an important impact on health care if QDs are successfully translated from the laboratory to the clinic.

It also shows emerging applications of QDs in targeted drug delivery and theranostics. Much research on QDs has been done on their use in imaging and diagnostics, but their application in personalized medicine, in which they are to be used to deliver drugs directly into particular cells or tissues, is relatively unexplored [29]. Further research in these areas of work should seek new methods and technologies through which to exploit the full potential of QDs for healthcare.

Practical Implications

In the near future, several practical implications following these findings will be relevant to QDs for health. First, purely biocompatible and low-toxicity QDs have to be developed in order to ensure safe integration into clinical practice. That is, researchers and healthcare professionals must be sure about the strict safety criteria required for new materials being used in medicine. Interest in QDs for both theranostics and personalized medicine is a fast-growing field, insinuating that such technologies could play a key role in the future of healthcare through more precise and effective treatments [30]. Application of QDs to the diagnostic and therapeutic fields may establish revolutionary changes in treating certain diseases, especially cancers, leading to better outcomes for patients.

This also demonstrates international collaboration in the area, and this is a factor making progress in QD research. As such, collaborative efforts between countries and institutions can expedite the process of developing new QD technologies, therefore fast-developing translational applications from research studies. Last but not least, the identification of emerging themes within the research provides a roadmap for future studies, thus guiding researchers into the most promising research areas on QDs. It is in these areas one could place much hope for the scientific community to continue stretching the bounds of what can be done with QDs in health, hence improving the quality of care and expanding the range of treatments available to patients.

## Conclusions

The bibliometric analysis done in this article underlined the significant growth and development in research into QDs in the health sector within the last two decades. QDs have come out as versatile tools in biomedical applications, with challenges related to their toxicity and biocompatibility. From the approach, a very strong global investigative network is noted, led by China, USA, and India in scientific contributions. It also identifies the emerging trends in research, particularly on aspects such as biosensing, nanotechnology, and targeted therapy that may potentially lead to further research. Despite the progress, continued innovation is essential to address remaining challenges and realize the potential role of QDs in improving healthcare outcomes. These findings, from the bibliometric analysis, inform useful insights for researchers and policymakers, pointing to future research priorities while continuing to strengthen international collaboration in this rapidly developing field.
